# Evolution of Tick Vaccinology Highlights Changes in Paradigms in This Research Area

**DOI:** 10.3390/vaccines11020253

**Published:** 2023-01-24

**Authors:** Agustín Estrada-Peña, José de la Fuente

**Affiliations:** 1 Department of Animal Health, Faculty of Veterinary Medicine, Miguel Servet, 177, 50013 Zaragoza, Spain; 2 Group of Research on Emerging Zoonoses, Instituto Agroalimentario de Aragón (IA2), 50013 Zaragoza, Spain; 3 SaBio (Health and Technology), Instituto de Investigación en Recursos Cinegéticos IREC-CSIC-UCLM-JCCM, Ronda de Toledo s/n, 13005 Ciudad Real, Spain; 4 Department of Veterinary Pathobiology, Center for Veterinary Health Sciences, Oklahoma State University, Stillwater, OK 74078, USA

**Keywords:** tick, vaccine, scientometric, scientific publications, citations, theme maps

## Abstract

Ticks and tick-borne diseases affect human and animal health worldwide. Although some tick-protective antigens have been identified and characterized, further research is needed for the development and application of effective anti-tick vaccines, which currently are unavailable for human protection. To study the trends and gaps in anti-tick vaccine approaches, herein we used scientometric analysis to evaluate several aspects of tick vaccinology. Co-authorship and citations networks pointed out two main research fronts, one focused on the laboratory protocols driving the recognition of candidate antigens and the other devoted to field experiments of protection against ticks. The analysis demonstrated the prominence of research in European countries on the topic. The scientometric approach allowed the recognition of isolated teams working casually on the topic, the lack of cooperation between middle- and low-income countries, and the need for sustained and integrated research. Most important, we identified a considerable lack of new candidates for vaccine development, as well as the participation of African and Asian countries. These results provide significant insights obtained from bibliographical analysis, suggest the strength and weaknesses in this field of research, and highlight new directions to advance in the development of effective vaccines for the control of tick infestations and tick-borne pathogens.

## 1. Introduction

Ticks are blood-feeding arthropod ectoparasites affecting human and animal health worldwide [1,2,3]. Ticks are vectors of pathogens and are associated with allergic pathologies, such as the alpha-gal syndrome [2,4]. Vaccines are the most effective and environmentally sound intervention for the control of tick infestations and pathogen infection and/or transmission [5]. Despite recent advances in the identification and characterization of candidate tick-protective antigens, only two anti-tick vaccines have been registered and commercialized for animal use [6]. Regarding human health, it is widely recognized that vaccination approaches focus more on tick-transmitted pathogens than on the control of the vectors [7,8], and therefore, no vaccines are available for use against ticks biting humans; most probably, this is because a candidate antigen allowing the rejection of the tick in the few hours after a bite (which is fundamental to avoiding the transmission of pathogens) has not yet been identified. Such prioritization of the pathogens promotes the atomization of the whole case (reservoirs–vectors–pathogens-health), and tick vectors remain only marginally addressed. A recent analysis of the gaps to integrate tick control methods into a “one health” strategy demonstrated that vaccination is, together with other practices related to environmental actions against ticks, a safer and more effective approach [9].

Focusing on the field of animal health, after decades of research, new candidates have not been registered for feasible tick control [10]. It is known that recent advances in “omics” technologies are a quantitative leap in the identification of tick-protective antigens [10]. We wanted to analyze the recent research on tick vaccines through a meta-analysis of the published scientific literature using a scientometric approach. Scientometrics is defined as a sub-field of bibliometrics focused on the measurement of the impact of scientific publications and research areas, with possible applications in advancing science and management [11]. We aimed to demonstrate how the topic has slowly evolved in the past 30 years, punctuated by sudden changes in paradigms quickly adhered to by most researchers in the field. This study not only aims to detect the main issues addressed by research on anti-tick vaccination since 1991 but also focuses on demonstrating the lack of connectivity of the main partners in this research field with important regions of the world, such as Africa and Asia, in which the largest economical losses occur due to ticks on livestock. We approached this study using both standard analysis of published references and a network-driven framework. Further data include details about the internationalization of teams and a proposal for moving forward. This study is not a review, nor is it aimed to explore the mechanism of action of the proposed approaches. We wanted to demonstrate how improvements in a field of research can be suggested, revealing gaps after exploring the hidden relationships in the scientific literature.

## 2. Materials and Methods

To track the evolution of the paradigms in anti-tick vaccines, we used records of published papers, including chapters of books and communications to congresses, if available in the search engines, obtained from the Web of Science (WoS) Core Collection, PubMed, and Scopus. Both the WoS and Scopus were accessed through the official accounts of the University of Zaragoza (Spain); PubMed is open for browse and query. The query was the same in every repository and consisted of the terms “vaccine OR Bm86 OR Akirin OR Subolesin OR 4D8 OR Q38” AND “Borrelia OR Anaplasma” OR “Babesia OR tick OR Ixodidae OR Argasidae” NOT “mosquito OR sandflies OR mite” and the topics “Immunology OR Parasitology”. The query was intended to capture papers related to the most prominent proteins used in tick control, as well as general vaccination strategies. It is important to note that some studies were revisionary works on the control of arthropods (i.e., including mosquitoes, sandflies, or mites). Since these studies generated noise in the query, making necessary a dedicated and long visual analysis for removal, the inclusion of these basic terms as “NOT” was enough to remove most of these manuscripts from the search strategy. However, a total of 20 review papers, related to tick-borne pathogens, ticks as parasites, control of ticks, etc., were included to check for their impact on the networks (see Table 1). For some of these documents, it is possible to obtain both the keywords, as submitted by the authors, or a special set of terms, called the “Keywords plus” that homogenize and enhance the original set of terms. Both sets of words were kept.

References were downloaded as Endnote-formatted files, and repeated papers in the queries to three repositories were removed. The content of each paper was visually checked by reading the abstract, looking for reviews and/or medical or veterinarian texts not directly related to the topic. The complete search and subsequent filtering produced a total of 274 documents (including papers in journals, congress communications, and book chapters; see Table 1 for details). The complete file of references used for this study is available as Supplementary Table S1. It is necessary to homogenize terms for a thorough bibliometric analysis, since the same term (i.e., the species of tick) may be used as either the common name or the Latin binomial. We used a file of synonyms prepared while conducting the preliminary tests of the study and after visual inspection of the keywords used by researchers. The word “Subolesin” prevailed over other names for the same antigen, such as “Akirin,” “4D8,” and “Q38” antigens. Other candidate antigens, not related to Subolesin or Bm86, such as calreticulin (CRT) and cathepsin L-like cysteine proteinase (CathL), were left as entered in the original references using the abbreviations. Additionally, all the ways of denominating the cattle tick, *Rhipicephalus microplus* or *Boophilus microplus* (but also “cattle tick,” “common cattle tick,” etc.), were converted into “microplus” after visual inspection of the abstracts.

We used the package “bibliometrix” [12] for R environmental programming [13]. After upload of the final Endnote-formatted file into the R environment, we addressed two categories of analysis, namely (i) analysis of sources, authors, and documents and (ii) analysis of conceptual, social, and intellectual structures. The former is a basic indication of the main journals publishing the type of papers under consideration, together with the trends in the time of the sub-topics of research in the field; the latter includes co-authorship and co-citations networks, other than maps showing the main geographical links among the co-authors in the field (see later for further explanations on networks).

For co-authorship and citations networks, we undertook a deeper analysis than the one produced by R. We used Gephi V0.92 (http://gephi.org, last accessed on 7 March 2022) to handle the networks of co-authors and co-citations, reading the files exported from R. A network is a set of nodes (either authors or publications) that are linked by edges displaying a “strength of connection.” In our context, such strength is either the number of times that a pair of authors co-authored publications or the number of times each publication has been cited by others. The strength of connections in the co-citation network allows the calculation of indexes of centrality. These indexes detect the relative impact of the papers in the network, thus pinpointing potential topics of interest for the community of researchers. Reviews were removed in this stage because they tend to be more cited, even if they do not report original significant advances in the field, resulting in a bias of the centrality indexes. Networks also created clusters of nodes detected by the Louvaine algorithm, running within Gephi. Clusters display either the authors of papers that tend to interact more frequently among them than with other authors or papers that belong to a sub-field of research (a category within the main topic).

We also developed thematic maps (also called strategic diagrams) resulting from the co-occurrence of keywords [14]. Complete details about the construction of thematic maps have been described previously [15]. Basically, the method checks for the co-occurrence of any pair of keywords, weighted by the times they appear alone, creates a network of co-occurrences, and calculates centrality and density values [14]. In our application, the plot is divided into four quadrants, called Niche themes, Motor themes, Basic themes, and Emerging or Declining themes [14,15]. Motor themes are in quadrant I (upper right) and are themes qualified as important pillars that shape the field of research. Niche themes in quadrant II (upper left) reflect highly developed but isolated topics of research. Emerging or Declining themes (quadrant III, lower left) are topics with weak development and that are highly marginal in the queried topics. Finally, Basic themes (quadrant IV, lower right) are less developed topics but still of importance to the field of study.

## 3. Results and Discussion

Table 1 includes the main results of the bibliographical query, further developed in this manuscript. A total of 94 sources produced 274 documents, with a total of 5619 unique references. The annual growth rate of publications on the topic (5.95) and the high average age of each document (more than 11 years) are of interest. This indicates a relative lack of growth on the topic, or, in other words, the adherence of research in the field to “old methods” with relatively few changes in paradigms.

As expected for tick vaccine research, the top journals with the highest number of publications were *Vaccine*, *Veterinary Parasitology*, *Experimental and Applied Acarology*, and *Ticks and Tick-Borne Diseases* (Supplementary Figure S1). Most but not all these journals have a hybrid format, allowing authors to pay for publication as “open access,” while another option includes a no-cost publication; in these cases, a subscription is necessary for reading the manuscript. Although some journals may wave article publication costs for certain middle-income countries, this is important for the publication by researchers belonging to poor-resource countries, since the most prominent journals publishing information about the target topic may be unaffordable for them in terms of both publishing and reading advances on the topic. 

**Figure 1:**
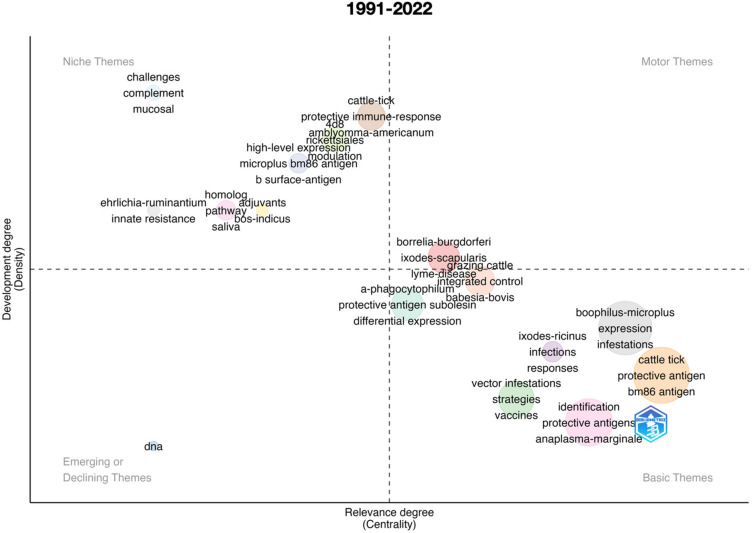
Thematic roles of the documents published on the topic “anti–tick vaccination” and obtained from a query of the literature in PubMed, Scopus, and Web of Science. The meaning of the four quadrants (defined as “themes”) is explained in the Section 2. The figure covers the complete period 1991–2022 and is analyzed in subsequent figures by time slices.

**Figure 2:**
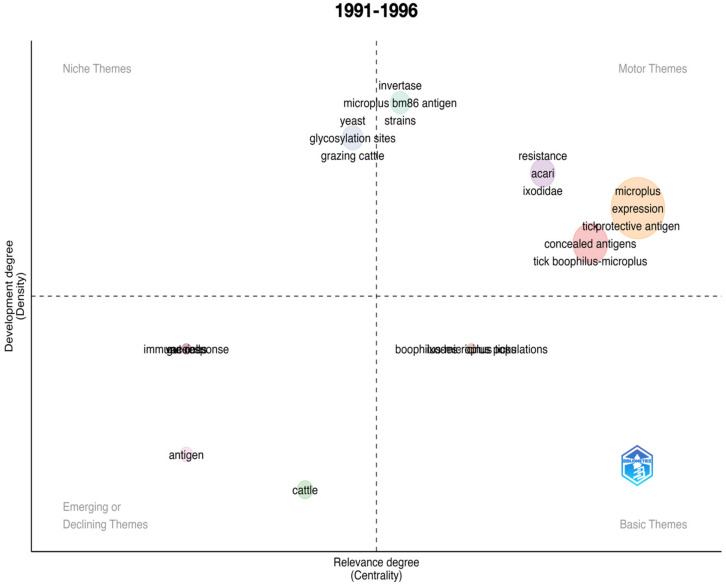
Thematic roles of the documents published on the topic “anti–tick vaccination” in the period 1991–2022.

**Figure 3:**
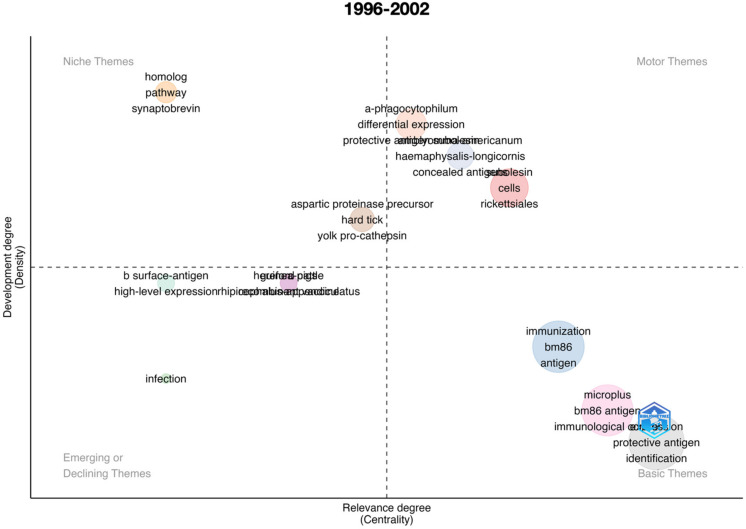
Thematic roles of the documents published on the topic “anti–tick vaccination” in the period 1996–2002.

**Figure 4:**
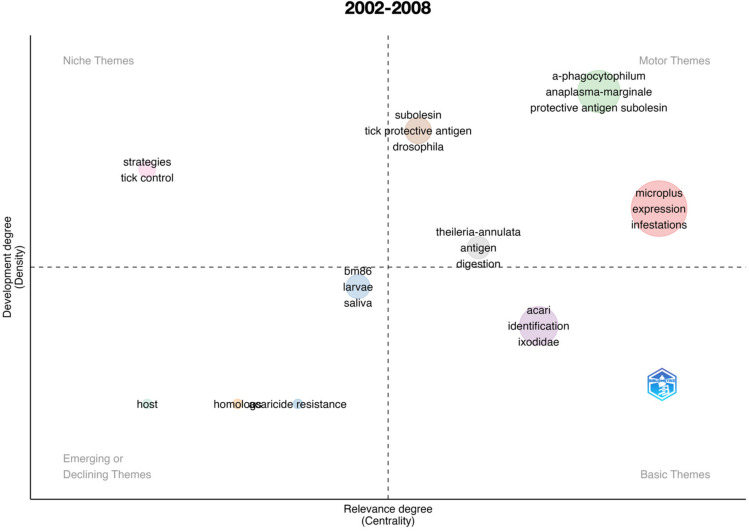
Thematic roles of the documents published on the topic “anti–tick vaccination” in the period 2002–2008.

**Figure 5:**
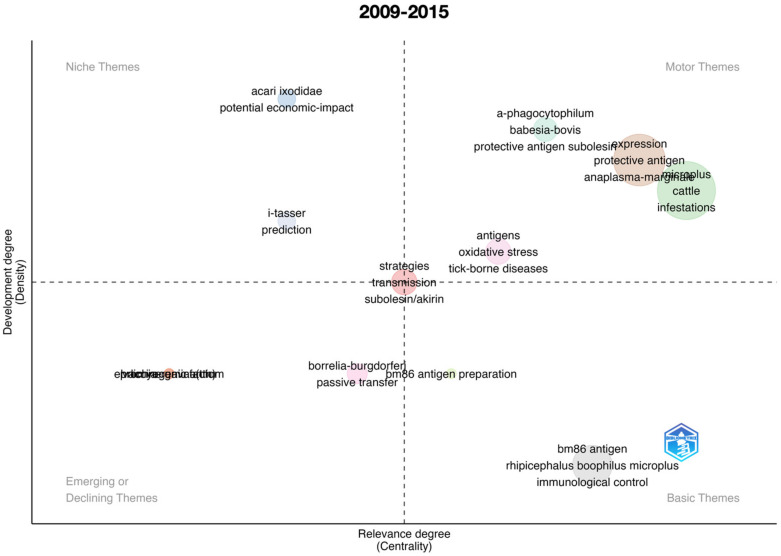
Thematic roles of the documents published on the topic “anti–tick vaccination” in the period 2009–2015.

**Figure 6:**
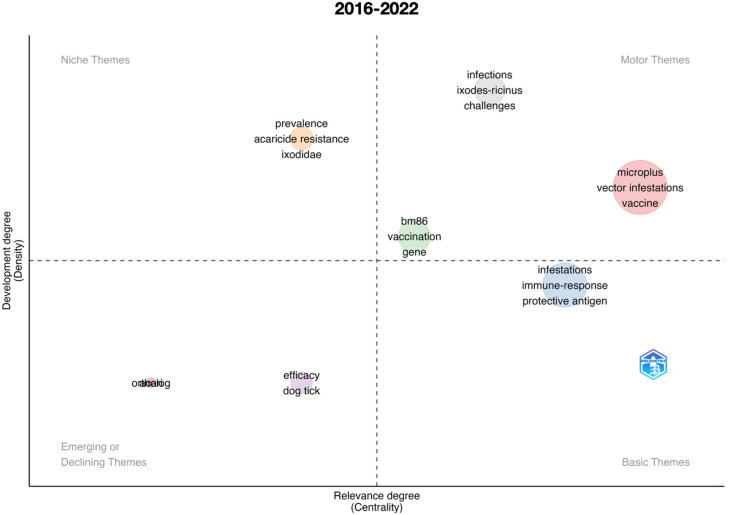
Thematic roles of the documents published on the topic “anti–tick vaccination” in the period 2016–2022.

An important impact of scientometric analyses is the evaluation of the evolution of the topic in time. Figure 1 summarizes the results for the complete period 1991–2022, further analyzed separately in Figure 2, Figure 3, Figure 4, Figure 5 and Figure 6 at slices of such period at a resolution of 6 years each; the latter procedure allowed a better evaluation of the “evolution of the topic.” Research on *R. microplus* and the vaccine-protective antigen Bm86 was the starting point in tick vaccine research [16]. This fact correlates with the appearance of the term “microplus” in Motor and Basic themes with high relevance and centrality indexes for the complete period from 1991 to 2022 (Figure 1). *Rhipicephalus microplus* has been the most tested species, most probably because of its invasive character and overall importance in animal health [17]. The themes related to one of the main antigens for tick control (and the one first discovered, Bm86) also appear as a Motor theme in 1991–1996 (Figure 2) and as Basic themes in 1996–2002 (Figure 3) and 2009–2015 (Figure 5) with increasing relevance. In other words, the research on vaccination against the tick *R. microplus* is a recurrent topic, even after a change in paradigms occurred with the finding of the Subolesin antigen as one of the main proteins currently used in vaccination experiments for tick control [18]. It is of importance to mention that most of the tests (laboratory or field derived) have been carried out on *R. microplus*, with a minor interest on other, locally important species. Themes related to the tick-protective antigen Subolesin began to appear in the relevant bibliography around the year 2002 and continued until 2015 as a Motor theme (Figure 3, Figure 4 and Figure 5). Our conclusion of this analysis is that the number of antigens targeted for tick control is low and that the interest of researchers has been balancing from one to another along the years because of limited development of new approaches.

Regarding tick-borne pathogens, *Anaplasma phagocytophilum* appeared as a Motor theme in 1996–2002, with growing relevance during 2009–2015. Interestingly, this is the only human pathogen of relevance, shared with wild animals, that has been most tested in this field of research, with lower attention to other prominent pathogens, such as rickettsiales or *Borrelia*, organisms that also affect human health. As mentioned before, we excluded vaccinology approaches to the control of ticks affecting humans, because preliminary tests of this study demonstrated a lack of context in the field. The lack of studies on ticks affecting humans is probably due to the mechanism of activity of some anti-tick vaccines allowing the tick to feed for a relatively short time before it is rejected [19]. This short time of tick feeding would be enough for the transmission of pathogens [20], and therefore, interest in the known candidate antigens may be low for their use regarding hypothetical protection in humans. However, other tick-borne pathogens affecting animal health (e.g., *Babesia* spp., *Theileria* spp., or *Anaplasma marginale*) have been already targeted for control using a vaccination against their main vector [21,22]. In the case of protozoans, such as *Babesia* or *Theileria*, the life cycle of the pathogen in the tick vector is different: the protozoan species must complete its life cycle before successful transmission from the tick to the animal, allowing a longer time for the vaccine to interact with the tick feeding.

**Figure 7:**
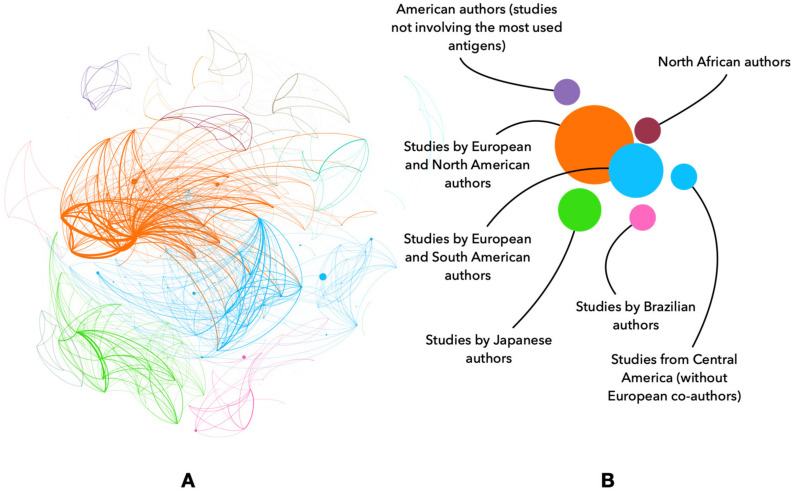
(**A**) Simplified view of the network of co-authors, displaying only nodes and links, with the labels removed to improve readability. The complete network with the names of the authors is available in Supplementary Material Figure S8 and allows on-screen zoom for improved reading. The simplified network is shown in (**A**); colors are clusters of co-authors who interact more among themselves than with other authors in the network. The meaning of the colors is given in (**B**), with a short explanation of the groups of authors included in each cluster. Other small clusters of the network not included in such schematized representation are anecdotical for the meaning of the network but can be explored in the complete network displayed in Supplementary Material Figure S8.

**Figure 8:**
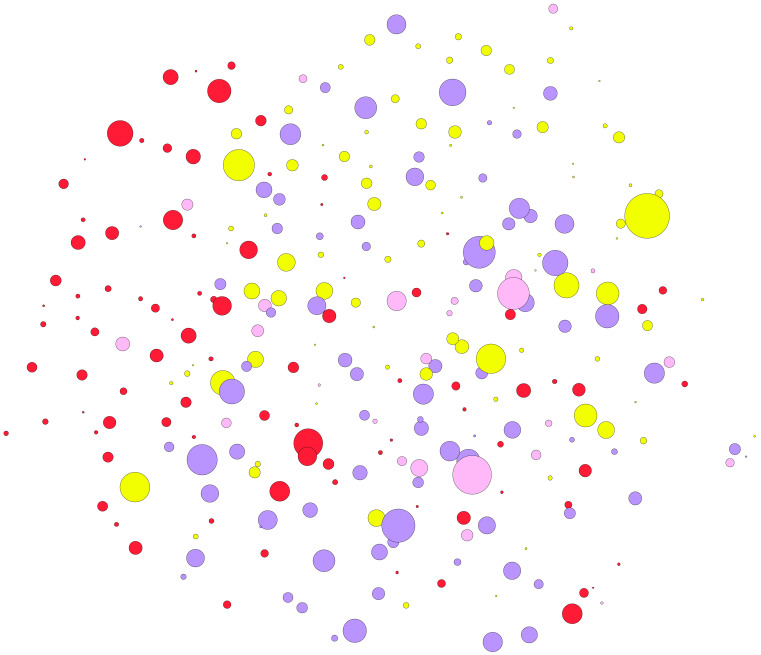
A simplified view of the network of citations, displaying only nodes and links, with the labels removed to improve readability. The complete network with the names of the authors is available in Supplementary Material S8 and allows on-screen zoom for improved reading. In the network, each node represents a document and each link represents a citation (e.g., the references included in each document). The size of the circle is proportional to the centrality of the document, or how many papers cite such specific document. Colors represent clusters, as sets of references that cite some papers more frequently than others. The red color represents the scientific literature on the topic mainly involved in laboratory studies; the yellow color refers mainly to documents dealing with field studies. Other categories are of difficult classification and are built up by studies that integrate both laboratory and field work.

The scientometric analysis also provided information about the authors’ production of scientific papers since 1991, the most relevant authors and the most highly cited papers, the top authors’ production over time, the importance of each country in the publications, and the collaboration among countries (Supplementary Figures S1–S6). We consider these charts are self-explained, and they do not require further elaboration. However, the network analysis of both co-authorship and co-citations provided information essential to understanding the strengths and weaknesses of the scientific community in this area (Figure 7 and Figure 8; Supplementary Material Figures S7 and S8). The results evidenced that most interactions are related to continued collaborations between scientists who are in close geographical vicinity or funded by continent-wide initiatives (i.e., European funds for research); these collaborations have some impact on the connection between groups in other regions. However, countries in Africa and Asia with a high incidence of tick-borne diseases are not well represented, except for some countries, such as India, China, South Africa, and Kenya. These results support the need to promote collaboration with scientists and institutions in “neglected” African and Asian countries (e.g., [23]) to translate scientific results into “one health” applications with health, economic, and social impact. Additionally, multi- and cross-disciplinary collaborations are rare, thus highlighting the need to promote these interactions to advance research in the development of novel interventions for the control of tick-borne diseases.

The network of co-authorship highlights that most significant findings in the field have been elaborated by few research teams (see the schematized Figure 7 and the complete network in Supplementary Material Figure S8). These results point commonly to a single or a few countries in Europe in which the development of high-technology research is commonly carried out, significantly ignoring the formation in these technologies in poor-resource countries. A second layer of co-occurring nodes is formed by representatives of South American countries, reflecting the interest in the application of these developments in the field, because these are the countries in which serious economic losses due to ticks and tick-borne diseases are commonly observed. This cluster does not include African or Asian countries, where the same problem exists, again reflecting the lack of involvement of these countries in the research on the topic. There is an interesting exception for South America, since another cluster of South American researchers was identified as authors of some few papers not linked with the previous set of authors. This may be because of the active search for other vaccination strategies (i.e., subunits of epitopes) carried out in some of these countries that remain out of the main flow of the topic [24]. Chinese, Japanese, and Brazilian authors formed clusters separated from the main network (Figure 7 and Supplementary Material Figure S8). The occurrence of the two first groups of researchers is anecdotical in comparison with the prominence of other countries.

The analysis of co-citations in the published scientific literature (Figure 7 and Supplementary Material S8) suggests two main research areas, with two main clusters related to basic investigations (displayed in red in the figure) and field applications (displayed in yellow). This is of interest since we could conclude that this field of research has two main aspects: the molecular research in the wet laboratory and the field experiments testing the candidate vaccines. Results indicated, however, that both sets of “approaches” are adhered to by several papers that link both facets of research (see different colors linking the two most prominent branches of research). However, there is yet a disappointingly low number of references addressing this challenging approach.

These results indicate that the research on vaccines for the control of cattle tick infestations has led vaccinology for ticks and tick-borne pathogens with Bm86-like antigens evaluated in different tick species and the discovery of new tick-protective antigens, such as Subolesin. Data for other antigens are purely anecdotical, and the tests on other species are rare and restricted to species of local interest. A coordinated approach to research and field tests is missing. The results also evidenced gaps in the need for collaborations with countries in Africa and Asia facing problems with ticks and tick-borne diseases probably due, among other factors, to limited resources for this research. It became evident that based on both co-citation and co-authorship networks, many researchers in countries with low funding support for these experiments are under-represented in the literature on the topic. Most important, the separated clusters identified in co-authorship networks are indicative of some teams addressing the research for a short period and then disappearing from the main flow of collaborations. This could be produced by the lack of interest of these researchers on the topic, by insufficient funding to continue the experiments, or by the low interest manifested by other teams in the approach explored in these studies.

## 4. Conclusions

A scientometric analysis can reveal trends and gaps in the research on vaccination against ticks affecting animal health. The results of this analysis demonstrated that the international collaboration regarding tick control by vaccination should be a must but currently lacks the active involvement of middle-income countries, considering that the largest economic losses due to ticks on livestock occur in low- or middle-income countries. However, active programs for the involvement of foreign researchers are short term and do not contribute to the in situ development of control strategies. The transfer of technology seems to be neglected in most cases, since the topic is recurrent in many countries. The consideration of other approaches (new candidate antigens), delivery methods, or the indirect control of animal tick-borne pathogens are occasionally addressed but lack regularity. This supports a side conclusion of our bibliometric analysis, indicating that efforts in the cooperation and transfer of technology to countries in which ticks are a substantial economic issue must be sustained in time. Not only field experiments can be developed there, but also technologically adequate facilities and formation of specialized personnel are a must if we aim for the control of ticks on livestock and transmitted pathogens.

## Figures and Tables

**Table 1:** Summary of information about the sources and papers used for this study. Left column indicates the items summarized in this study.

Time span (years)	1991–2022
Sources (journals and books)	94
Documents	274
Annual growth rate %	5.95
Document average age	11.5
Average citations per document	29.86
References	5619
DOCUMENT CONTENTS	
Keywords plus (ID)	644
Author’s keywords (DE)	564
Authors	1019
Authors of single-authored docs	7
Single-authored documents	12
Co-authors per document	7
International co-authorships	48.91%
DOCUMENT TYPES	
Article	228
Article; book chapter	4
Article; early access	1
Article; proceedings paper	14
Editorial material	1
Letter	1
Meeting abstract	3
Proceedings paper	2
Review	20

## Data Availability

All the data generated and used in his study are available in the Supplementary Material section.

## Supplementary Materials

The following supporting information can be downloaded at https://www.mdpi.com/article/10.3390/vaccines11020253/s1: Figure S1: Most relevant sources publishing papers related to the field of anti-tick vaccination, including the number of papers published in each journal. The inset shows the “core sources” or the four journals publishing the highest proportion of papers related to the topic; Figure S2: Most relevant authors publishing papers related to the field of anti-tick vaccination, including the percentage of papers recovered by the query of bibliographical repositories and analyzed in this study; Figure S3: Most globally cited documents that include topics of tick vaccination, according to the analyzed references. The DOI of each document is included close to the Y axis for interested readers; Figure S4: A timeline indicating the productivity of each author (in the list of the most prominent ones). The size of each circle indicates the number of published papers; the color of each circle represents the compared productivity of the researcher according to the publication of other papers on the topic in the same year (in %); Figure S5: A world map of collaborations among the co-authors of the papers on the target topic; Figure S6: An overview of the single- (SCP) or multi-country (MCP) origin of the authors of papers devoted to anti-tick vaccination in the period 1991–2022. The X axis indicates the number of documents per country in the period 1991–2022; Figure S7: The complete view, with labels for the names of each author, of the co-authorship network shown in Figure S7; Figure S8: The complete view, with labels for the names of the first author of each paper of the co-citation network shown in Figure S8; Supplementary Table S1: The complete list of references used in this study.
