# Supplementary material for: Evolution of Tick Vaccinology Highlights Changes in Paradigms in This Research Area
Source: Vaccines (Basel). 2023 Jan 24;11(2):253. doi: 10.3390/vaccines11020253 (PMC9962838; doi:10.3390/vaccines11020253)
Supplement: Supplementary file 1 [file vaccines-11-00253-s001.zip › Supplementary Figure S1.pdf]

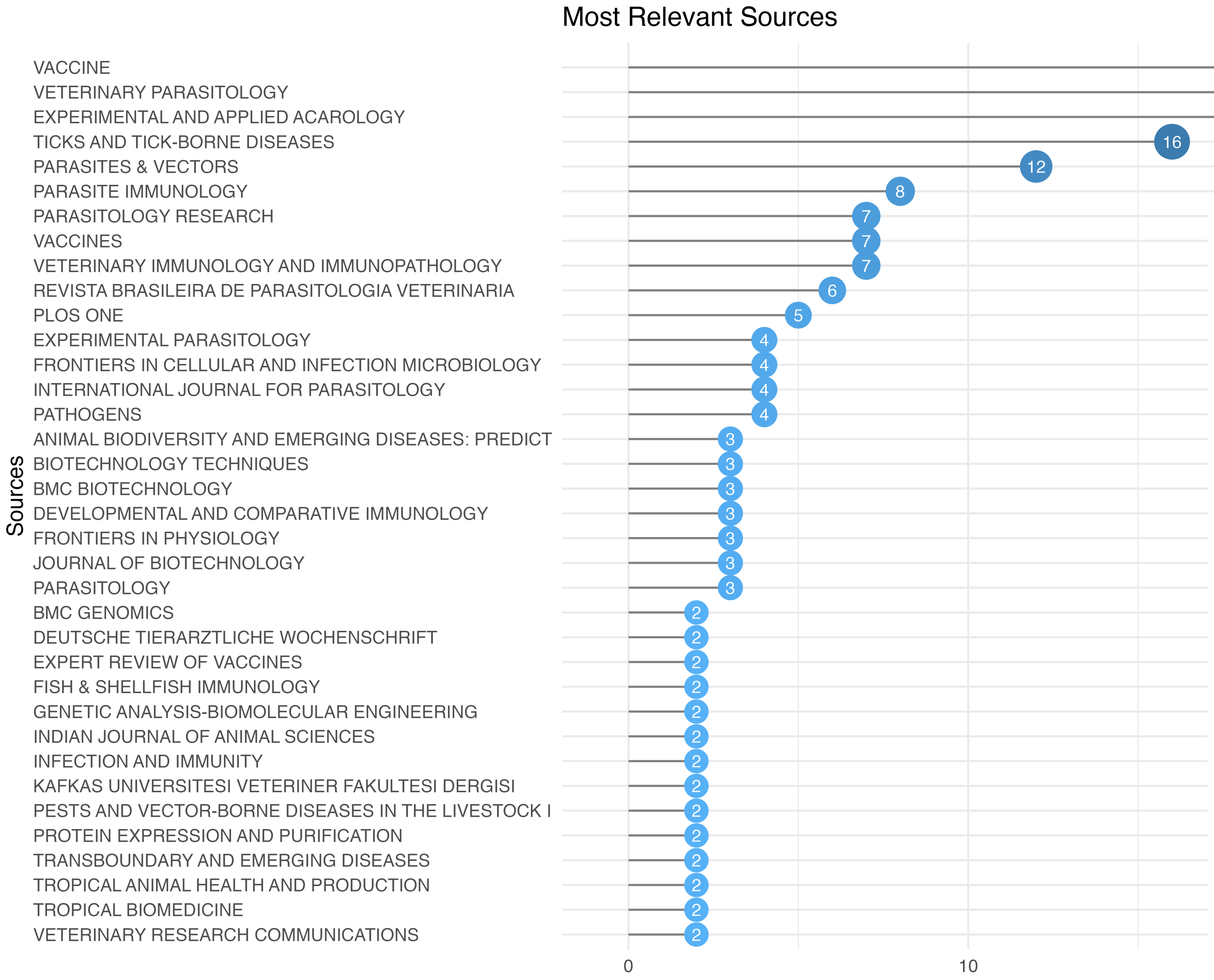
Most Relevant Sources
Sources
VACCINE
VETERINARY PARASITOLOGY
EXPERIMENTAL AND APPLIED ACAROLOGY
TICKS AND TICK-BORNE DISEASES
PARASITES & VECTORS
PARASITE IMMUNOLOGY
PARASITOLOGY RESEARCH
VACCINES
VETERINARY IMMUNOLOGY AND IMMUNOPATHOLOGY
REVISTA BRASILEIRA DE PARASITOLOGIA VETERINARIA
PLOS ONE
EXPERIMENTAL PARASITOLOGY
FRONTIERS IN CELLULAR AND INFECTION MICROBIOLOGY
INTERNATIONAL JOURNAL FOR PARASITOLOGY
PATHOGENS
ANIMAL BIODIVERSITY AND EMERGING DISEASES: PREDICT
BIOTECHNOLOGY TECHNIQUES
BMC BIOTECHNOLOGY
DEVELOPMENTAL AND COMPARATIVE IMMUNOLOGY
FRONTIERS IN PHYSIOLOGY
JOURNAL OF BIOTECHNOLOGY
PARASITOLOGY
BMC GENOMICS
DEUTSCHE TIERARZTLICHE WOCHENSCHRIFT
EXPERT REVIEW OF VACCINES
FISH & SHELLFISH IMMUNOLOGY
GENETIC ANALYSIS-BIOMOLECULAR ENGINEERING
INDIAN JOURNAL OF ANIMAL SCIENCES
INFECTION AND IMMUNITY
KAFKAS UNIVERSITESI VETERINER FAKULTESI DERGISI
PESTS AND VECTOR-BORNE DISEASES IN THE LIVESTOCK I
PROTEIN EXPRESSION AND PURIFICATION
TRANSBOUNDARY AND EMERGING DISEASES
TROPICAL ANIMAL HEALTH AND PRODUCTION
TROPICAL BIOMEDICINE
VETERINARY RESEARCH COMMUNICATIONS
39
26
18
16
12
8
7
7
7
6
5
4
4
4
4
3
3
3
3
3
3
3
2
2
2
2
2
2
2
2
2
2
2
2
2
2
0
10

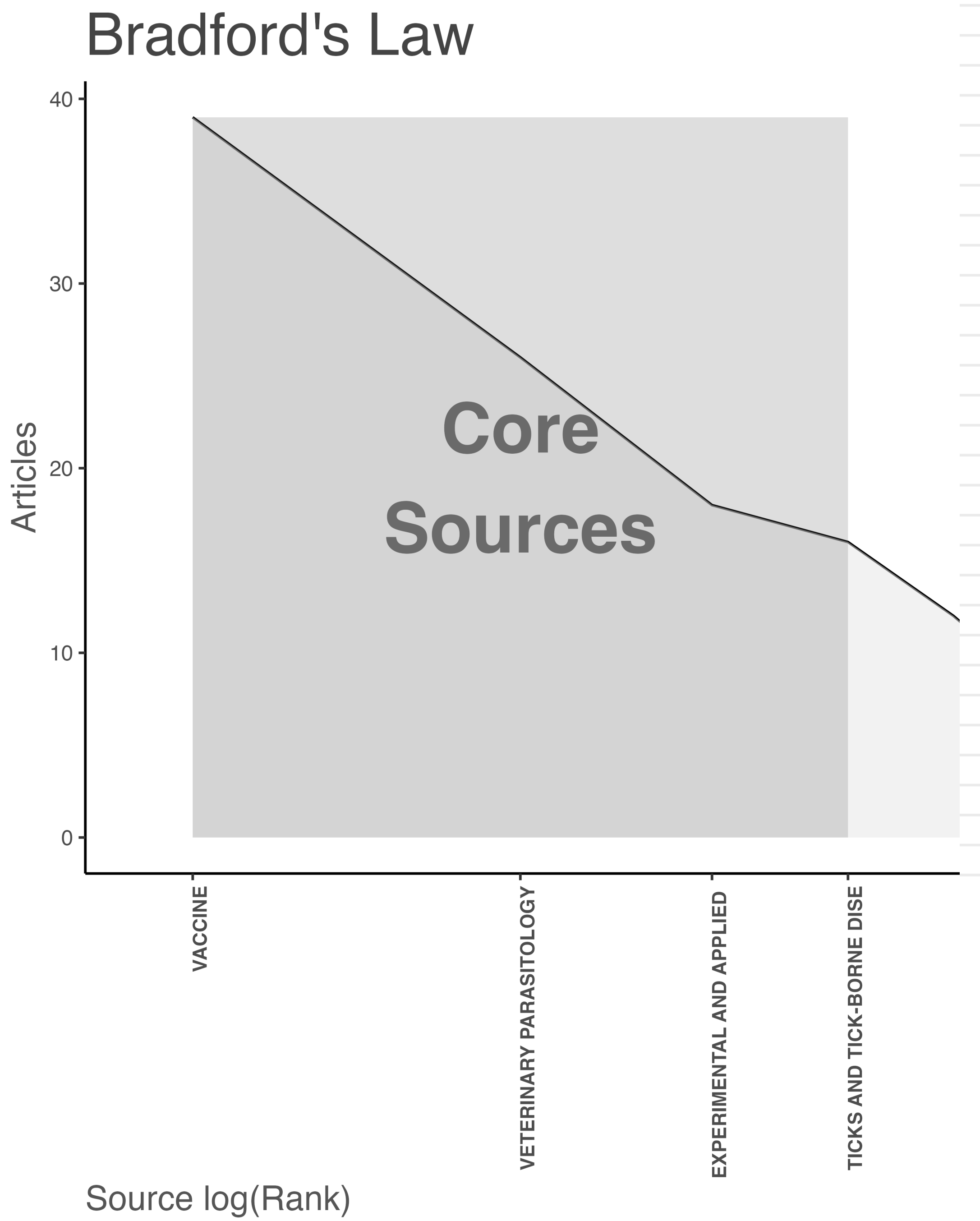
Bradford's Law
Articles
40
30
20
10
0
Core
Sources
VACCINE
VETERINARY PARASITOLOGY
EXPERIMENTAL AND APPLIED
TICKS AND TICK-BORNE DISE
Source log(Rank)
