# Supplementary material for: Evolution of Tick Vaccinology Highlights Changes in Paradigms in This Research Area
Source: Vaccines (Basel). 2023 Jan 24;11(2):253. doi: 10.3390/vaccines11020253 (PMC9962838; doi:10.3390/vaccines11020253)
Supplement: Supplementary file 1 [file vaccines-11-00253-s001.zip › Supplementary Figure S6.pdf]

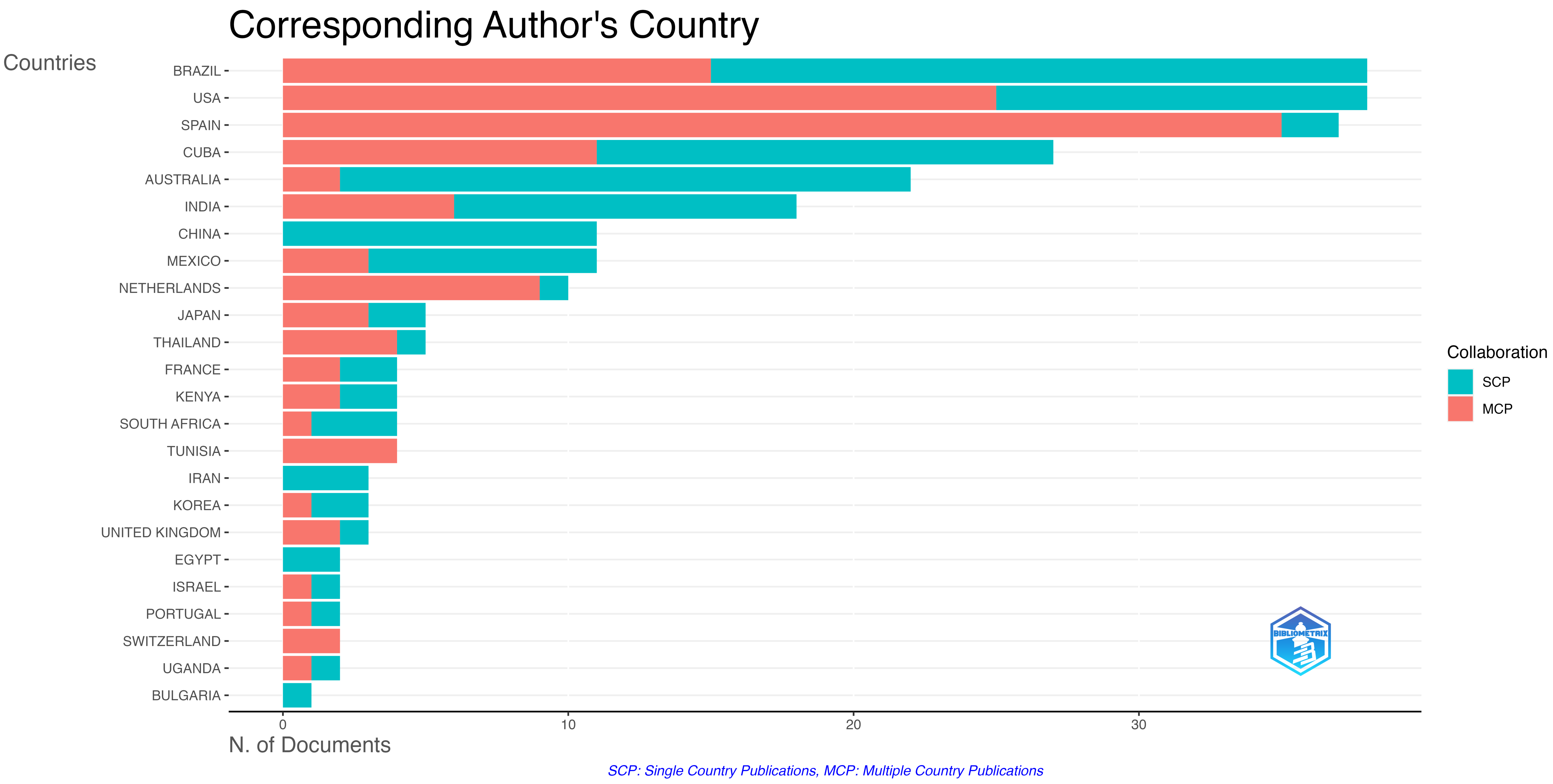
Corresponding Author's Country
Countries
BRAZIL
USA
SPAIN
CUBA
AUSTRALIA
INDIA
CHINA
MEXICO
NETHERLANDS
JAPAN
THAILAND
FRANCE
KENYA
SOUTH AFRICA
TUNISIA
IRAN
KOREA
UNITED KINGDOM
EGYPT
ISRAEL
PORTUGAL
SWITZERLAND
UGANDA
BULGARIA
0
10
20
30
N. of Documents
Collaboration
SCP
MCP
SCP: Single Country Publications, MCP: Multiple Country Publications
