# Supplementary figures and images for: Evolution of Tick Vaccinology Highlights Changes in Paradigms in This Research Area
Source: Vaccines (Basel). 2023 Jan 24;11(2):253. doi: 10.3390/vaccines11020253 (PMC9962838; doi:10.3390/vaccines11020253)

Most Relevant Authors

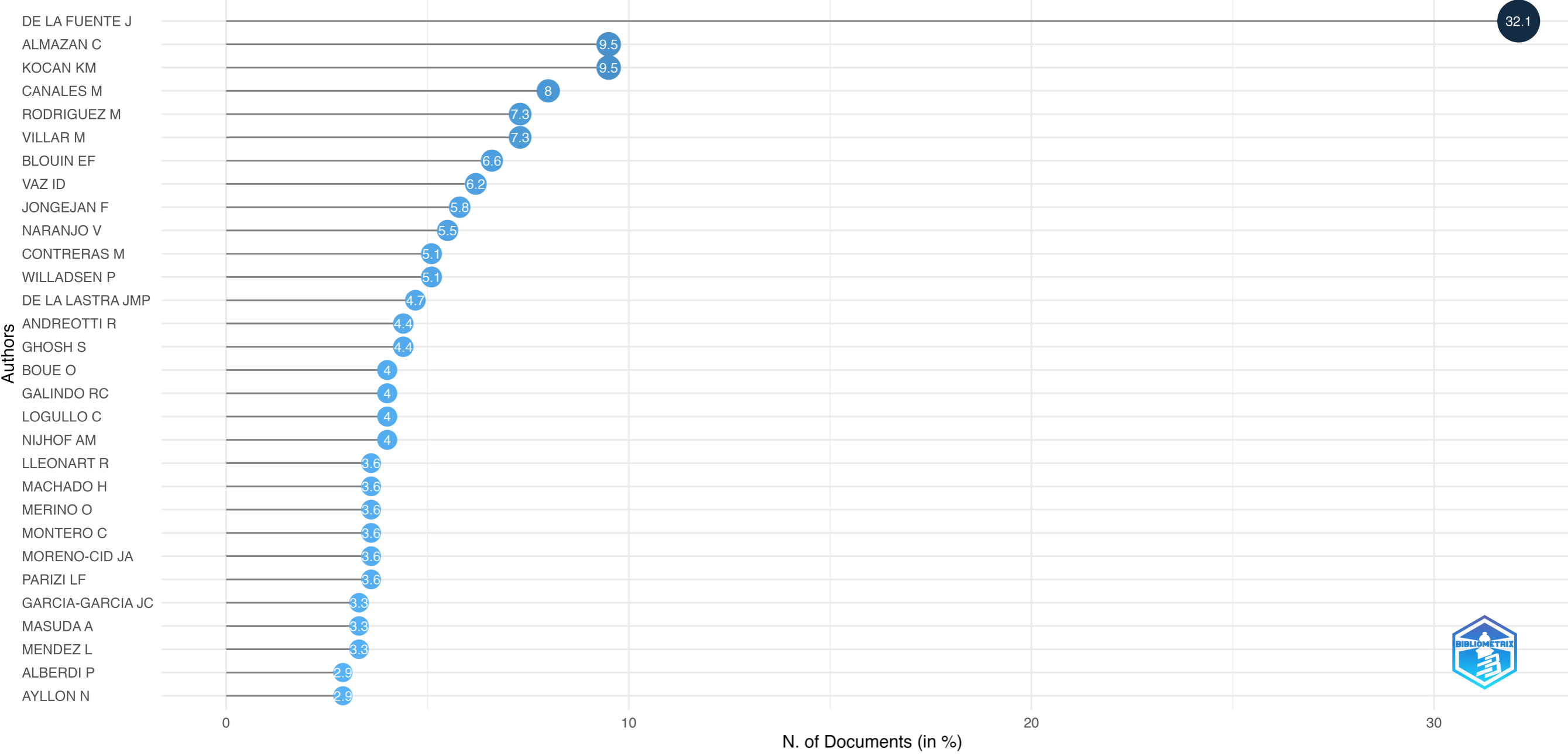

Supplement: Supplementary file 1 [file vaccines-11-00253-s001.zip › Supplementary Figure S2.pdf]

Most Global Cited Documents

Documents

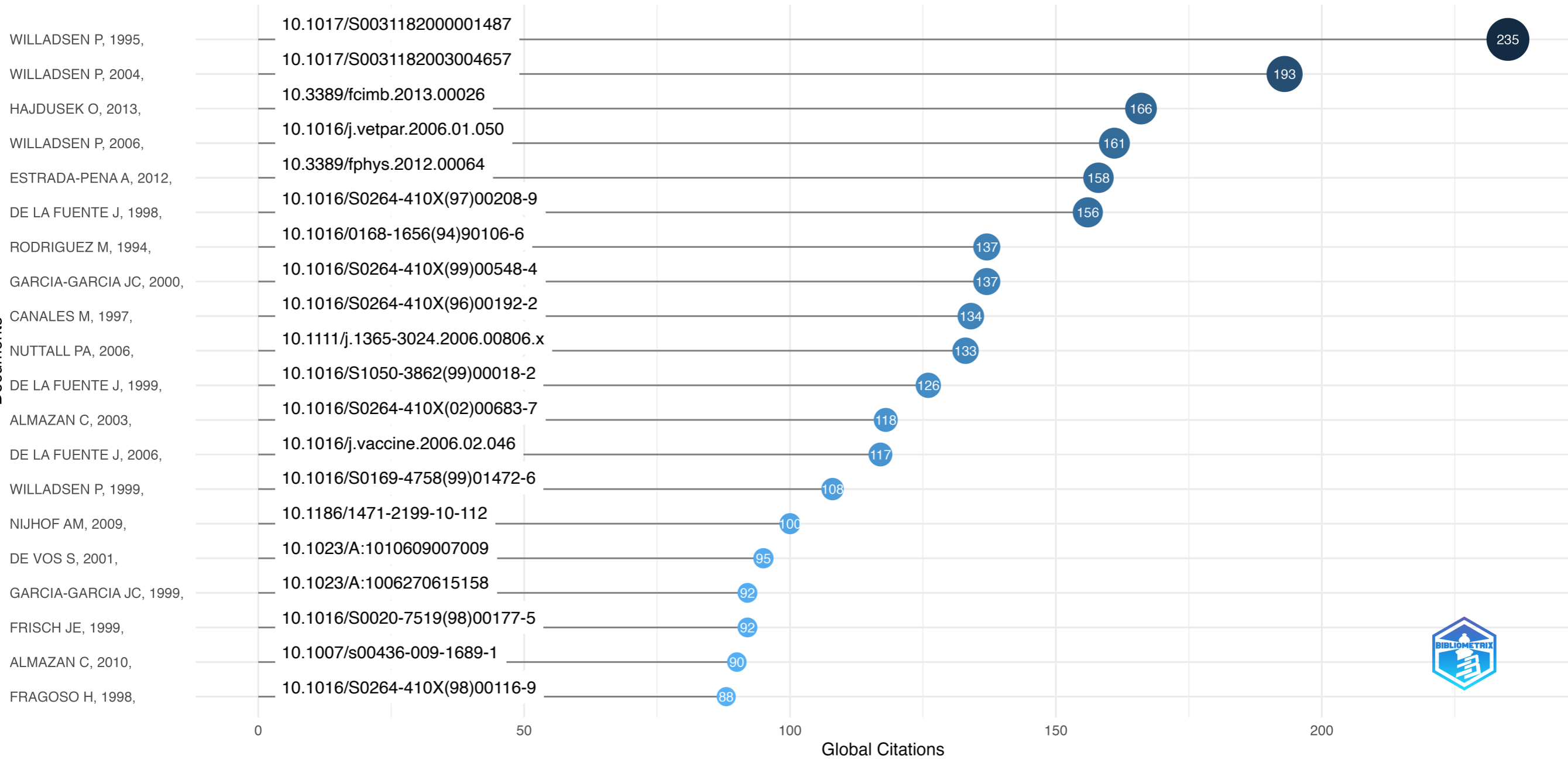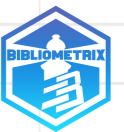

Supplement: Supplementary file 1 [file vaccines-11-00253-s001.zip › Supplementary Figure S3.pdf]

# Top-Authors' Production over Time

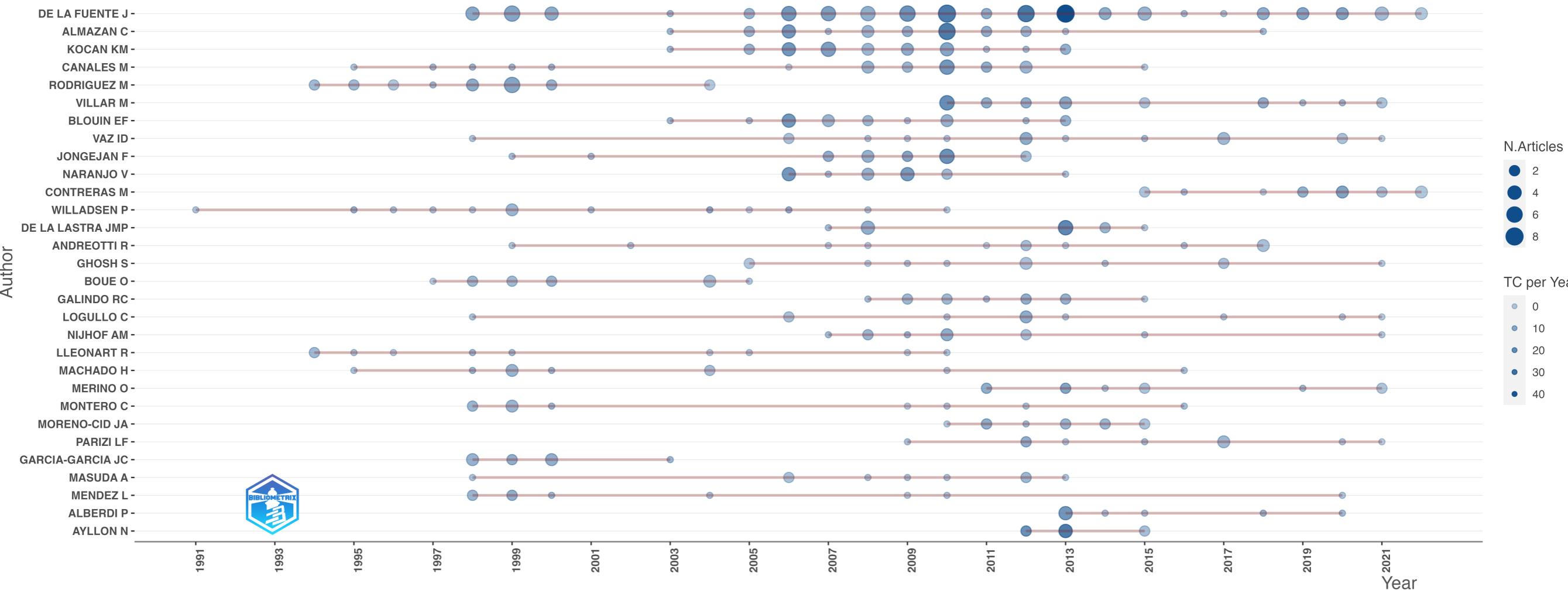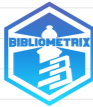

Supplement: Supplementary file 1 [file vaccines-11-00253-s001.zip › Supplementary Figure S4.pdf]

# Country Collaboration Map

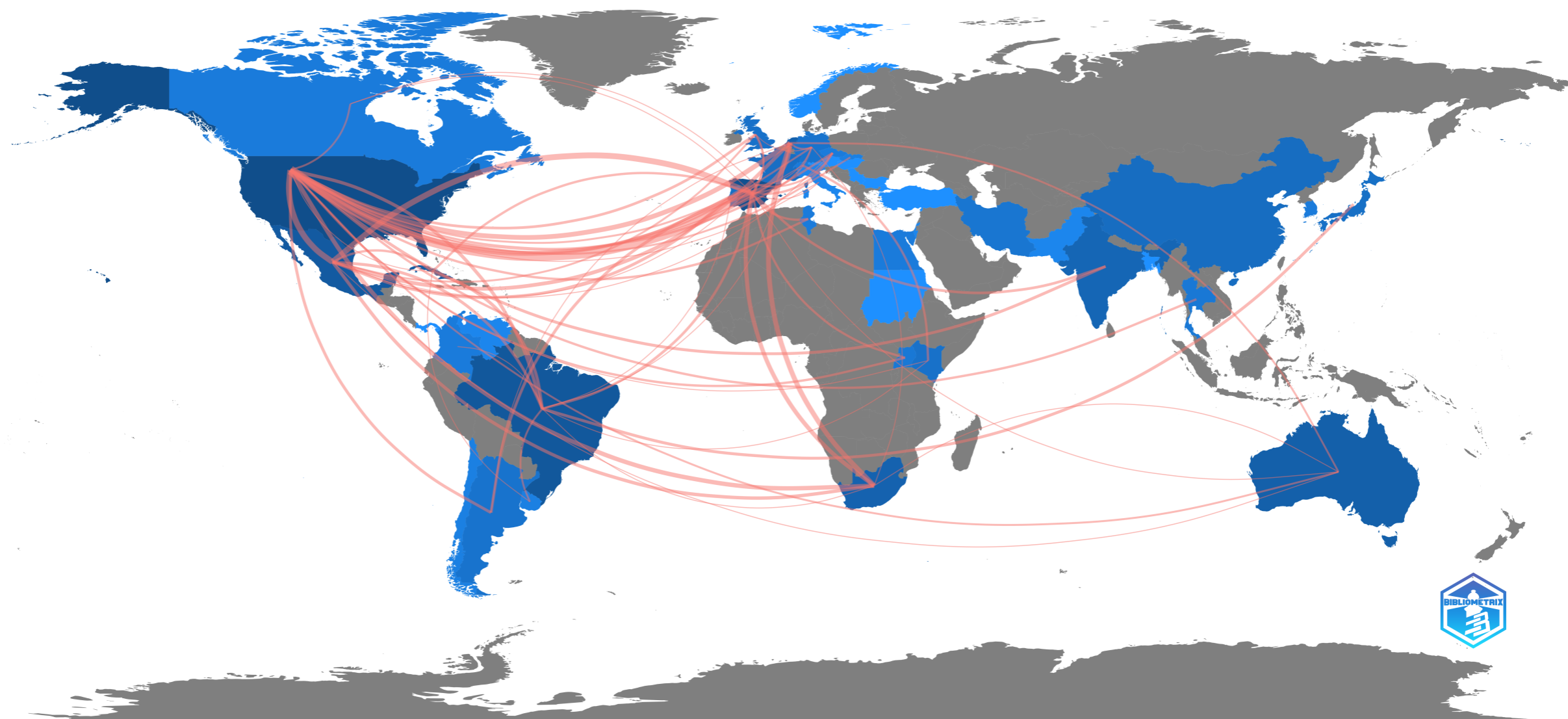

Supplement: Supplementary file 1 [file vaccines-11-00253-s001.zip › Supplementary Figure S5.pdf]

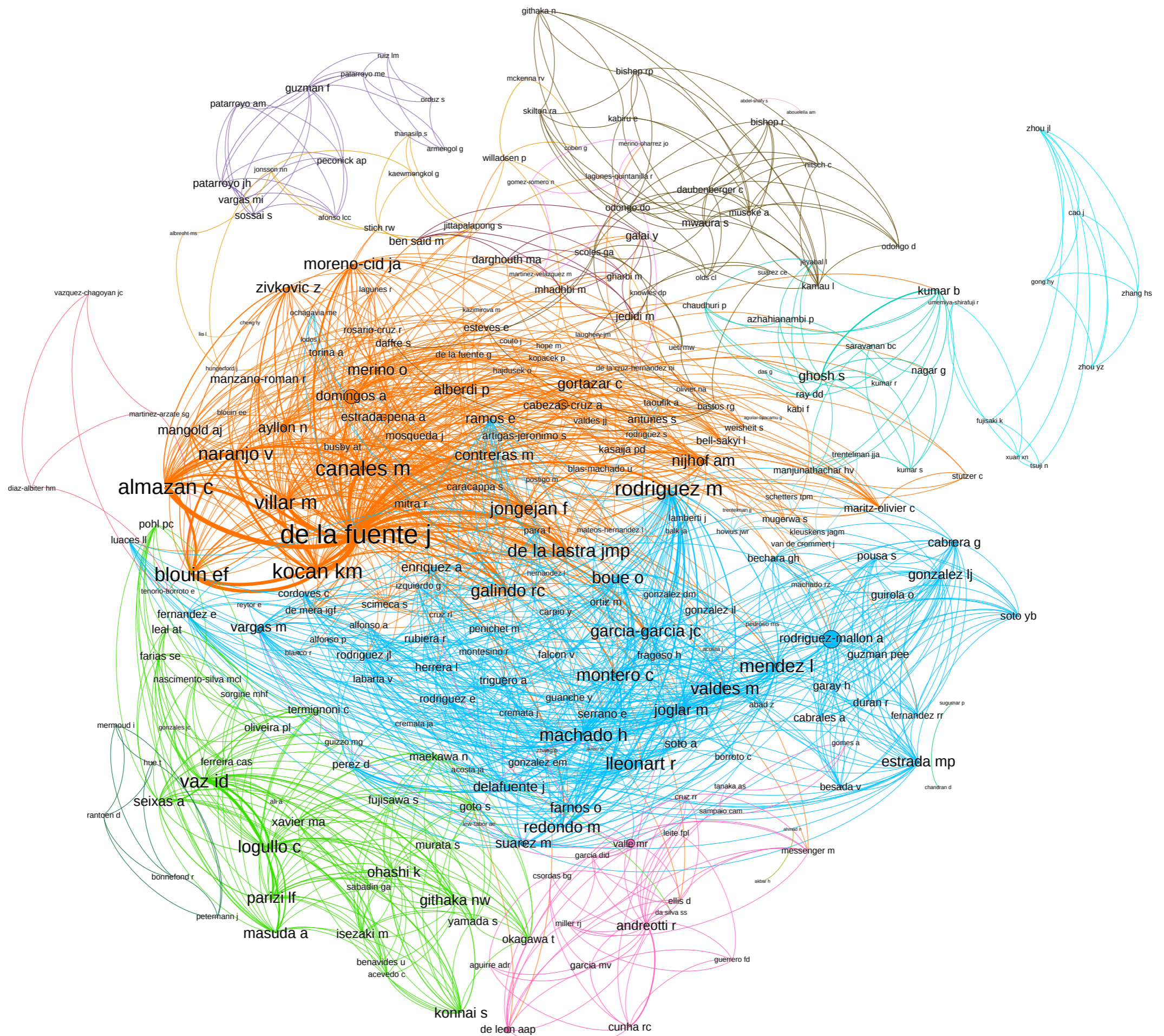

Supplement: Supplementary file 1 [file vaccines-11-00253-s001.zip › Supplementary Figure S7.pdf]

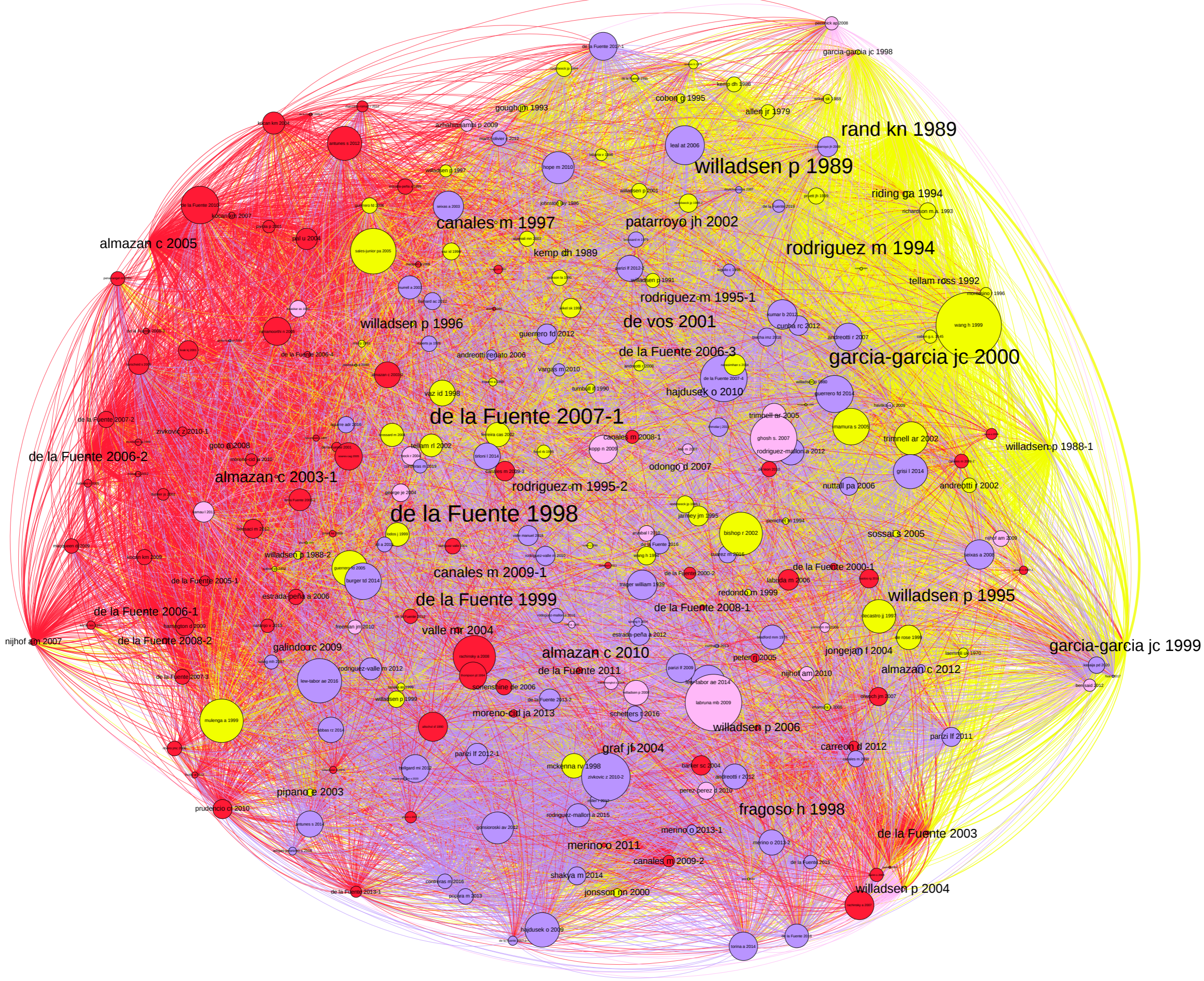

Supplement: Supplementary file 1 [file vaccines-11-00253-s001.zip › Supplementary Figure S8.pdf]
